# Decellularized Pig Kidney with a Micro-Nano Secondary Structure Contributes to Tumor Progression in 3D Tumor Model

**DOI:** 10.3390/ma15051935

**Published:** 2022-03-04

**Authors:** Shuangjia Yang, Le Zheng, Zilong Chen, Zeren Jiao, Tianqing Liu, Yi Nie, Yue Kang, Bo Pan, Kedong Song

**Affiliations:** 1 State Key Laboratory of Fine Chemicals, Dalian R&D Center for Stem Cell and Tissue Engineering, Dalian University of Technology, Dalian 116024, China; yangshuangjia@mail.dlut.edu.cn (S.Y.); lezheng@mail.dlut.edu.cn (L.Z.); zilongchen@mail.dlut.edu.cn (Z.C.); liutq@dlut.edu.cn (T.L.); 2 Artie McFerrin Department of Chemical Engineering, College Station, Texas A&M University, Texas, TX 77843-3122, USA; jiaozeren@tamu.edu; 3 Zhengzhou Institute of Emerging Industrial Technology, Zhengzhou 450000, China; 4 Key Laboratory of Green Process and Engineering, Institute of Process Engineering, Chinese Academy of Sciences, Beijing 100190, China; 5 Department of Breast Surgery, Cancer Hospital of China Medical University, 44 Xiaoheyan Road, Dadong District, Shenyang 110042, China; 6 Department of Breast Surgery, The Second Hospital of Dalian Medical University, 467 Zhongshan Road, Shahekou District, Dalian 116023, China

**Keywords:** extracellular matrix, derived scaffolds, pig kidney, micro-nano secondary structure, tumor model

## Abstract

In spite of many anti-cancer drugs utilized in clinical treatment, cancer is still one of the diseases with the highest morbidity and mortality worldwide, owing to the complexity and heterogeneity of the tumor microenvironment. Compared with conventional 2D tumor models, 3D scaffolds could provide structures and a microenvironment which stimulate native tumor tissues more accurately. The extracellular matrix (ECM) is the main component of the cell in the microenvironment that is mainly composed of three-dimensional nanofibers, which can form nanoscale fiber networks, while the decellularized extracellular matrix (dECM) has been widely applied to engineered scaffolds. In this study, pig kidney was used as the source material to prepare dECM scaffolds. A chemical crosslinking method was used to improve the mechanical properties and other physical characteristics of the decellularized pig kidney-derived scaffold. Furthermore, a human breast cancer cell line (MCF-7) was used to further investigate the biocompatibility of the scaffold to fabricate a tumor model. The results showed that the existence of nanostructures in the scaffold plays an important role in cell adhesion, proliferation, and differentiation. Therefore, the pig kidney-derived matrix scaffold prepared by decellularization could provide more cell attachment sites, which is conducive to cell adhesion and proliferation, physiological activities, and tumor model construction.

## 1. Introduction

Breast cancer, lung cancer, and colorectal cancer are the three most prevalent cancers among women, which account for 50 percent of all new diagnoses. Among those, breast cancer alone accounts for 30 percent of diagnosed cancer in women [1]. In the United States, 1 in 8 (13%) women can be diagnosed with invasive breast cancer in their lifetime. By 2019, the number of new breast cancer cases in the United States had surpassed 250,000, and 3.8 million American women have a history of breast cancer so far [2], Therefore, there is always a need for an in vitro 3D environment for breast cancer tumor model construction to better study the biological behavior of tumor cells and provide a platform for the screening of tumor drugs. 

The 2D culture environment method is a routine method for studying malignant breast cells and stromal cells in vitro, but this will lead to the loss of 3D structure and can negatively affect cell interaction and function, resulting in inconsistent results from the test in vivo [3]. In addition, 2D cell culture cannot replicate the various characteristics of solid tumors in vivo and their resistance to drugs. This results in a large number of ineffective drugs tests being tried directly on animals, which not only leads to the excessive use of animals but also prolongs the time for drug discovery [4]. There is always a need to find a medium to connect two-dimensional cell culture with animal experiments in vivo, so the establishment of a three-dimensional cell culture model in vitro has been put on the agenda. Various scaffolds based on biomaterials have been widely used in three-dimensional cell culture models. The biomaterials used to make scaffolds may be natural polymers such as alginate [5], collagen [6], gelatin [7], fibrin [8], and albumin [9], or synthetic polymers such as polyvinyl alcohol [10], polyethylene glycol [11], and polyacrylamide [12]. Scaffolds made from different materials have different properties. For natural polymer materials, although they have a good biocompatibility and are conducive to cell adhesion and proliferation, their mechanical properties are poor, with a relatively higher degradation rate. Synthetic polymer materials have good mechanical properties and are easily processed. Their structure and properties can be modified and regulated as needed, which makes them more suitable for mass production. However, synthetic polymer materials still lack cell action sites and have poor cell adhesion.

Decellularization-derived matrix scaffolds have become a replacement platform for tumor tissue engineering due to their biomechanical properties and tissue-specific extracellular matrix (ECM) composition [13]. An ECM is an acellular three-dimensional polymer network composed of collagen, proteoglycan/glycosaminoglycan, elastin, fibronectin, laminin, and several other glycoproteins [14,15]. It is highly dynamic because it is continuously deposited, remodeled, and degraded during development until it matures to maintain tissue homeostasis. Therefore, its composition varies with tissue change according to specific requirements [16]. ECM protein can guide the differentiation of embryonic stem cells and is an important factor that affects cell behavior during organogenesis. In particular, if the ECM protein structure is very similar to the ECM structure of the target tissue in vivo, the differentiation to the target tissue can be improved [17,18]. ECMs are a component of the cellular microenvironment, mainly composed of three-dimensional nanofibers. The fiber diameter is generally between 50 and 500 nm [19]. The nano-level fiber network formed plays an important role in cell adhesion, proliferation, and differentiation [20]. Different sources of decellularized extracellular matrixes (dECMs) have been developed, most of which are derived from patients or animals and are accessible. Instead of experiments on living animals, decellularization only requires parts of pathological tissues or fresh animal tissues from market sale. The kidney is highly heterogeneous, with complex vascular structures and many functional structures. Its highly complex microstructure and basement membrane composed of different proteins which can support at least 26 different cells are closely aligned [21,22]. In addition, because the evolution of pigs is similar to that of humans, there are similarities in physiological activities and organ size [23]. Therefore, pig kidney is chosen as the 3D environment for constructing breast cancer tumor models.

Solid tumors grow in vivo and their development process is the result of the constant interaction between tumor cells and the microenvironment. The kidney has a highly complex microscopic structure with nearly 30 types of cells, a network of blood vessels, a basement membrane composed of different proteins which support the adhesion of at least 26 different types of cells, as well as many functional structures. In addition, breast tumors are metastatic and may metastasize to the kidney. Thus, in order to better study the biological behavior of tumor cells (atypia, diffusion, and metastasis [24]) and anti-tumor drugs screening, this study uses porcine renal tissue as the main raw material to construct an acellular-derived matrix scaffold. Due to the limited mechanical properties of the ECM, chemical crosslinking technology was used to further modify and prepare the required pig kidney acellular-derived matrix scaffold. On this basis, the feasibility of constructing a tumor model was further explored by inoculating breast cancer (MCF-7) cells into the derived scaffold. By comparing the physical properties of acellular matrix scaffolds before and after crosslinking, the effect of crosslinking on the scaffolds and whether it denatures the acellular matrix were investigated. The biocompatibility of the scaffold and the size of the tumor ball were studied by laser confocal and scanning electron microscopy to evaluate the construction of breast cancer tumor models. The two-dimensional environment was also constructed to compare the difference in cell growth with the three-dimensional method. In this study, the effects of a secondary microstructure on tumor progression and cell adhesion were investigated, aiming to provide a platform with a higher biocompatibility for drug screening and basic cancer research.

## 2. Materials and Methods

### 2.1. Materials

Human breast cancer MCF-7 cells were purchased from Shanghai Zhongqiao Xinzhou Biotechnology Co., Ltd. (Shanghai, China). The adipose tissue was provided by the Affiliated Hospital of Dalian Medical University (Dalian, China). Human adipose tissue-derived stem cells (ADSCs) were isolated from subcutaneous adipose tissue obtained from liposuction surgery performed on a 32-year-old woman. The process strictly adhered to the requirements of the Helsinki Declaration with the patient’s consent. MCF-7 and ADSCs were cultured in a humidified incubator at 37 °C with 5.0% CO_2_ and Dulbecco’s improved Eagle medium (DMEM) containing 10% fetal bovine serum. Sodium dodecyl sulfate (SDS) was purchased from Shenyang WanLei Technology Co. (Shenyang, China), LTD. N-(3-dimethylaminopropyl)-N’-ethyl carbonimide hydrochloride (EDC), N-hydroxysuccinimide (NHS), and 2-(N-morpholino) ethane sulfonic acid (MES) were purchased from Beijing Balinway Technology Co. (Beijing, China), LTD. DMEM and fetal bovine serum (FBS) were purchased from Gibco (Carlsbad, CA, USA). Calcein-AM, propyl iodide (PI), and Hoechst 33,342 were all purchased from Calbiochem (San Diego, CA, USA). A Cell Counting Kit-8 (CCK-8) was obtained from Selleck.cn (Shanghai, China).

### 2.2. Preparation of Acellular Porcine Kidney Matrix Scaffold

Fresh pig kidneys were purchased from the market, washed with clean water until the water had no obvious blood color, and then refrigerated at −20~−30 °C for 10~14 h. After freezing, the renal pelvis and fibrous capsule were removed, and the remaining kidneys were cut into small pieces of 10~16 cm^3^ in volume. The kidney block was bathed in phosphate (PBS) buffer and washed several times. The treated renal lumps were put into the 0.5~1 wt.% sodium dodecyl sulfonate (SDS) solution and agitated under low-speed for 48~72 h. At room temperature, the acellular matrix was soaked and washed in phosphate (PBS) buffer solution 6~10 times, and the solution was changed every 20 min. The matrix was frozen in a refrigerator at −20~−30 °C for 3~5 h and then freeze-dried for 14~19 h to obtain a porous kidney-derived matrix scaffold. The kidney-derived matrix scaffolds were then crosslinked for 36~48 h in ethanol solution containing 50 mmol/L 1-(3-dimethylaminopropyl)-3-ethyl carbonimide hydrochloride (EDC), 50 mmol/L N-hydroxysuccinimide (NHS), and 50 mmol/L 2-(N-morpholino) ethanesulfonic acid (MES). After crosslinking, the decellularized matrix scaffolds were cleaned with PBS buffer solution 5~6 times. Finally, the pig kidney decellularized scaffolds after cleaning were freeze-dried for another 19~24 h to obtain the crosslinked pig kidney-derived matrix scaffolds.

### 2.3. Analysis of the Micro-Nano Structure of the Porcine Kidney-Derived Decellularized Matrix Scaffold

The nanoscale structure of the pig kidney-derived decellularized matrix scaffold was observed by a Quanta 450 tungsten filament scanning electron microscope (FEI company, Hillsboro, OR, USA). The specific steps were as follows: 5 pig kidney cells-derived matrix scaffolds were cut into 3 mm × 3 mm × 1 mm blocks and fixed to the slides by conductive adhesive. The samples were then observed under different magnification after dust removal and spray gold operations. At last, the software Image-Pro Plus (IPP) (6.0 Version, Media Cybernetics Company, Rockville, MD, USA) was used to investigate the nanoscale structure quantitatively. Briefly, the scale in each SEM image was saved as a new spatial calibration. By measuring length and distance based on the above scale length, the fiber length can be automatically displayed. The fiber structure at different parts of each bracket was measured to calculate the average aperture.

### 2.4. Decellularization Efficiency Test

Scanning electron microscopy (SEM, SU1510, Hitachi High Technologies, Tokyo, Japan) was used to observe the microstructure, pore size, and vascular structure of the pig kidney matrix after decellularization and crosslinking. In addition, histological staining analysis was performed on the pig kidney matrix before and after decellularization. For the sample treatment, the samples were fixed by 2.5% glutaraldehyde, dehydrated through different concentrations of ethanol (50%, 70%, 80%, 90%, 100%), embedded by paraffin, and sliced into the thickness of 3 mm for hematoxylin and eosin staining (H&E), as well as Masson trichromatic staining.

### 2.5. Physical Performance Test of Pig Kidney Matrix Scaffold

The mechanical properties of the extracellular matrix were optimized by chemical crosslinking. In this study, the properties of the porcine renal decellularized matrix scaffolds, such as water absorption, porosity, compression modulus, infrared spectrum, contact angle, and thermal stability, were compared before and after the crosslinking, and the denaturation of the scaffold was also discussed.

#### 2.5.1. Water Absorption Rate

To compare the water absorption of the pig nephritic-derived matrix scaffolds before and after crosslinking, the dense surface of the kidney-derived matrix scaffold was excised and then the dry weight of each sample (n = 9) was measured as m1. Cut scaffold blocks were then placed in a 24-well plate filled with PBS buffer and let stand for 12 h. Scaffolds were then taken out, wiped of the moisture using absorbent paper, and weighted as m2. Multiple groups of each material were tested and the average value was taken as the experimental result. The water absorption rate of the scaffold can be calculated as

$$W\left(\%\right)=\frac{\;\left(m_{2}-m_{1}\right)}{m_{1}}\times 100\%$$

#### 2.5.2. Porosity

A volumetric flask with a volume of 10 mL was chosen and filled with ethanol, and was weighed as *W*_1_. The scaffold weighing *W_S_* was immersed into ethanol inside the volumetric flask and then placed in a vacuum dryer to drain the air inside the scaffold. After the ethanol was filled into the pore structure of the scaffold, the volumetric flask was filled with ethanol again and weighed as *W*_2_. The sample that had been filled with ethanol was taken out and the remaining ethanol and the volumetric flask was weighed as *W*_3_. The porosity of the scaffold can be calculated as

$$\Phi =\frac{\;\left(W_{2}-W_{3}-W_{S}\right)}{\left(W_{1}-W_{3}\right)}\times 100\%$$

#### 2.5.3. Contact Angle Detection

The differences in the contact angle of the decellularized matrix scaffold (n = 6) in pig kidney before and after crosslinking were compared. A drop (6 μL) of water was injected on the flat sample surface with a microsyringe and the droplet was filmed with a high-speed camera and the contact angle of the droplet was calculated by software.

#### 2.5.4. Infrared Spectrum Detection

Several kidney-derived decellularized matrix scaffolds (n = 9) before and after crosslinking were taken, and the dense outer membrane on the surface of the scaffolds was removed and then crushed after liquid nitrogen treatment [25]. The potassium bromide tablet method was used to analyze the processed scaffolds by infrared spectroscopy. In order to obtain the spectrum, a resolution of 4 cm^−1^ and a spectral wave number range of 4000 to 500 cm^−1^ were used to compare the spectral differences of the kidney-derived matrix scaffolds before and after crosslinking.

#### 2.5.5. Compressive Modulus Test

The mechanical properties of the decellularized pig kidney scaffolds (n = 9) before and after crosslinking were measured on a universal experimental machine [26]. Some scaffold materials before and after crosslinking were cut smooth with a certain thickness. The loading speed of longitudinal compression was 1 mm/min, and the maximum pressure was 50 N. The force-displacement data were obtained directly from the test results. The compression modulus of decellularized pig kidney scaffold can be expressed as

$$E=\frac{\sigma }{\varepsilon }=\frac{Y/AB}{\left(L-X\right)/L}$$

where *E* indicates the compression modulus; *Y* indicates the pressure load during the test; *A*, *B*, and *L* indicate the length, width, and height of the scaffold, respectively; and *X* indicates the compression depth under pressure load.

#### 2.5.6. Thermal Stability Analysis

The thermogravimetric (TGA) (METTLER TOLEDO, Greifensee, Switzerland) and differential scanning calorimetry (DSC) analyses (METTLER TOLEDO, Greifensee, Switzerland) were performed to compare the thermal stability of the scaffold derived from porcine kidney before and after crosslinking. Experimental conditions were as follows: the scaffold was ground into powder after being treated with liquid nitrogen, the temperature was increased at the rate of 10 °C/min under nitrogen protection, and the temperature increased from 25 °C to 600 °C. TA Universal Analysis 2000 thermal analysis software (5.0.1 Version, TA Instruments, New Castle, PA, USA) was used for data analysis.

### 2.6. Biocompatibility

ADSCs were cultured in DMEM containing 10% fetal bovine serum [27]. The culture medium was changed every three days. The biocompatibility of the pig renal decellularized matrix scaffolds was studied by the inoculation of ADSCs on the scaffolds. The scaffold treatment was as follows: the crosslinked pig renal matrix scaffolds were cut into small pieces with a volume of 5 mm × 5 mm × 2 mm, soaked in 75% alcohol, and placed on the ultra-clean table for overnight ultraviolet sterilization. The cells were immersed in PBS for two hours before inoculation, and the liquid was changed every 1 h. The scaffold was then placed in a 24-well plate and a double-sided inoculation method was adopted. A total of 20 μL of ADSC suspension with a cell density of 2 × 10^5^ cells/mL was taken and inoculated on the scaffold. After 1, 3, 5, 7, and 10 days, the cells were stained with 2 μmol/L calcein-AM, 4 μmol/L PI, and 5 μg/L Hoechst 33,342, incubated in an incubator at 37 °C for 30 min, and then observed and photographed under a confocal laser microscope.

### 2.7. Construction of Breast Cancer Tumor Model 

To compare the proliferation and cell morphology of breast cancer (MCF-7) cells in two- and three-dimensions, the biocompatibility of MCF-7 cells on the pig renal scaffolds, cell permeability, and tumor sphere size were studied.

#### 2.7.1. Growth Status of Breast Cancer Cells (MCF-7) on the Kidney Matrix Scaffold

The growth of MCF-7 cells on the pig renal matrix scaffolds was studied. A total of 20 μL MCF-7 cell suspension with a density of 4 × 10^6^ cells/mL was inoculated on the pig kidney scaffold and cultured for 3, 5, 7, 10, 14, and 21 days. A total of 2 μmol/L calcein-AM, 4 μmol /L PI, and 5 μg/L Hoechst 33,342 mixed staining solution was added and incubated at 37 °C for 2 to 3 h. The images were observed and photographed under a laser confocal microscope.

In addition, the proliferation of MCF-7 cells in the 2D and 3D environments was investigated. The cell activity was quantitatively analyzed by the CCK-8 method. In a 24-well plate, 20 μL of MCF-7 cell suspension was used for inoculation on the pig kidney scaffold. The cell density was 5 × 10^5^ cells/mL. The same number of MCF-7 cells were also inoculated in a 24-well plate for culture as the control group for 3D cell culture. Cell activity was measured on days 1, 3, 5, and 7 (n = 5). CCK-8 (10% v/v in DMEM) was added and incubated in an incubator at 37 °C for 3 h. Then, 100 μL of reaction solution was transferred to a 96-well plate. The optical density (OD) value at 450 nm was measured by a microplate reader (Varioskan Flash, Thermo Fisher, Waltham, MA, USA).

#### 2.7.2. Permeability of Breast Cancer Cells (MCF-7) on the Porcine Kidney Matrix Scaffold

The permeability of MCF-7 cells at different depths (10 μm, 40 μm, 100 μm) on the pig renal scaffolds was investigated. A total of 20 μL of cell suspension with a cell density of 1 × 10^6^ cells/mL was inoculated on the pig kidney scaffold. After 3, 7, and 21 days of culture, 2 μmol/L calcein-Am, 4 μmol/L PI, and 5 μg/L Hoechst 33,342 complex dye solution was added and incubated in an incubator at 37 °C for 3 h, and then observed under a confocal laser microscope. Fluorescence staining was used to locate the locations of living cells and nuclei to investigate the distribution of cells in the scaffold. Layer-by-layer scanning was carried out along the Z-axis of the structure. The image scanning interval was set as 10 μm, and the 3D reconstruction of the obtained multi-layer scanning image showed the distribution and infiltration of cells in the scaffold.

#### 2.7.3. The Size of Tumor Spheres in Breast Cancer 

Inverted microscopy, fluorescence microscopy, and scanning electron microscopy were used to analyze the size of the edge and inner tumor sphere of the pig kidney scaffold [28]. A total of 20 μL of cell suspension with a cell density of 4 × 10^6^ cells/mL was inoculated on the pig kidney scaffold. After 3, 5, 7, 10, 14, and 21 days of culture, the tumor spheres were observed and photographed under an inverted microscope, fluorescence microscope, and scanning electron microscope, respectively. The size of the tumor spheres was quantitatively analyzed using IPP software.

#### 2.7.4. Enzyme-Linked Immunosorbent Assay (ELISA)

On the 3rd, 7th, and 14th day of culture, the cells were centrifuged at 2500 rpm for 20 min to collect the supernatant of MCF-7 cells cultured on 2D or 3D renal scaffolds. The supernatant was stored at −20 °C before the experiment. According to the manufacturer’s instructions, the expressions of hypoxia-inducible factor-1α (HIF-1α) and breast cancer susceptibility protein 1 (BRCA1) were observed in the 96-well plate. The reference sample and test sample were properly diluted in advance. The OD value was measured at a 450 nm wavelength with a microplate reader. The HIF-1α and BRCA1 concentrations were calculated according to the standard curve.

### 2.8. Statistical Analysis

All experimental results were expressed as mean ± SD (standard deviation). The statistical significance of the differences between groups was assessed using a one-way ANOVA, and then the t-test analysis was performed using OriginPro (V9.0, OriginLab Corporation, Northampton, MA, USA). When *p* < 0.05, the test result was considered to have significant difference.

## 3. Results and Discussion

### 3.1. Decellularization Efficiency of the Porcine Kidney Matrix

**Figure 1:**
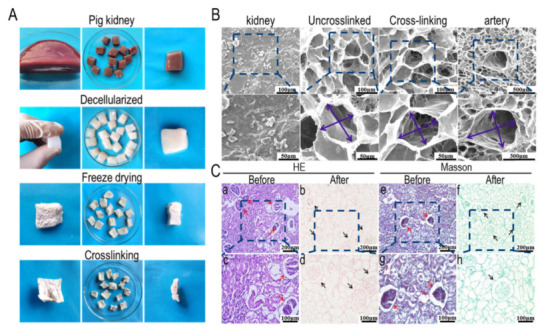
Decellularization efficiency of porcine kidney tissues. (**A**) Macrostructure of pig kidney matrix before decellularization, after decellularization and after crosslink; (**B**) SEM analysis of pig kidney matrix, uncrosslinked dECM, crosslinked dECM,and vascular structure of dECM; (**C**) H&E and Masson staining. (**a**,**b**) represent H&E staining of procine kidney matrix before and after decellularization, respectively. (**c**,**d**) represent Masson staining of procine kidney matrix before and after decellularization, respectively. (**e**), (**f**), (**g**) and (**h**) are magnification images of (**a**), (**b**), (**c**) and (**d**). scale: 200 μm (top), 100 μm (under).

Figure 1a shows the whole process of the pig renal decellularized matrix scaffold preparation. The porcine kidney matrix is elastic and appears light red before decellularization, and changes from light red to transparent after decellularization. When a certain pressure was applied on the decellularized matrix, the shape of the pig kidney matrix did not change dramatically, indicating that the pig kidney matrix had good mechanical properties after decellularization, which was also reflected in the subsequent compression modulus detection results. To improve the mechanical properties of the decellularized matrix, the matrix was treated by EDC/NHS, which could crosslink the amino group with the carboxyl group. EDC/NHS is non-toxic and the intermediate could be easily removed. In addition, scaffolds crosslinked by EDC/NHS exhibited a better biocompatibility. After crosslinking, the volume of the scaffold was slightly reduced after the secondary freeze-drying.

The micromorphology of the pig kidney matrix after different process steps is shown in Figure 1b. In tissue engineering, the key factor affecting the performance of scaffolds is the pore structure of the scaffolds. Therefore, in this experiment, the microstructure of the matrix scaffolds prepared was further observed by a scanning electron microscope. It can be seen from the figure that the scaffolds have abundant interconnected porous structures, which are conducive to intercellular contact and mass transfer. The microstructural surface of a pig kidney is very dense, which indirectly indicates the difficulty of the acellular process. Although there was no significant difference between the uncrosslinked- and the crosslinked-derived matrix scaffolds, further observation showed that the two were different in terms of micromorphology. The scaffold diameter of the pig kidney matrix without crosslinking was randomly distributed, with a large aperture range and some structures destroyed, and the aperture range was 132~363 μm. The matrix scaffold after crosslinking had a uniform pore size distribution and complete structure, which may be due to the repair of the scaffold structure during the crosslinking process and the reconnection of amide bonds damaged during the acellular process. The pore size range was 127~238 μm. The favorable pore structure of the scaffold provides sufficient space for cell proliferation and differentiation, which can improve the mass transfer ability between the pores of the scaffold and is therefore conducive to waste transfer and nutrition transport, and provides a balanced growth environment for the cell [29]. In the figure, we can see the complete vascular structure of the matrix scaffold, with the diameter of the vessels ranging from 927 to 1383 μm. In addition, the experimental results before and after decellularization showed that the effect of decellularization was outstanding, and the pore structure of the scaffold was well preserved.

Figure 1c shows Masson and H&E histological staining before and after decellularization in the pig kidney matrix. Ali et al. found that glomerular and tubular structures could be reserved after appropriate decellularization, which turned out to be red by H&E staining, and cells disappeared completely [30]. The decellularized extracellular matrix (dECM) showed intact pore structure and glomerulus, while cells were invisible, indicating that decellularization was successful. As reported, collagen and glycosaminoglycan (GAG) were abundant within native tissues, which are main components of ECM and provide support for cell growth and cell invasion [31]. Masson staining showed that the collagen structure of the extracellular matrix was preserved well and the complex collagen network retained after decellularization could contribute to cell adhesion [32]. The arrows indicate the glomerular structure. There was no obvious damage to the glomerular structure before and after decellularization, but only the corresponding red cell structure was removed. In addition to the glomerular structure, the structure of other parts was also intact, so the effect of decellularization was significant.

### 3.2. Microstructure and Elemental Analysis of the Pig Kidney-Derived Matrix Scaffold

**Figure 2:**
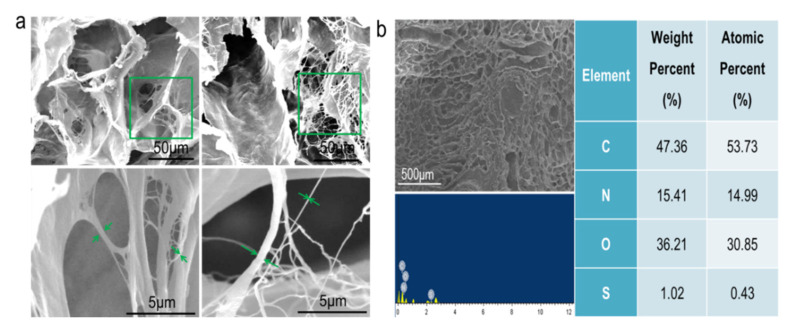
Microstructure analysis of pig kidney matrix scaffolds. (**a**) Reticular structure of pig kidney-derived matrix scaffold nanofibers; scale: 50 μm (top), 5 μm (under). (**b**) Element analysis of pig kidney-derived matrix scaffold.

The existence of nanostructures plays an important role in cell adhesion, proliferation, and differentiation. ECM is a component of the cell microenvironment, mainly composed of three-dimensional nanofibers, which can form nanoscale fiber networks [33,34,35]. It was observed by scanning electron microscope that the pig kidney matrix scaffold was composed of many fiber networks showing a honeycomb appearance. This result is consistent with what Hussein et al. reported, as well as the result of H&E and Masson staining, providing a supportive structure for cell growth [36]. The fiber network at different locations was further enlarged (green arrow) and the thickness of the fiber was measured using IPP software, ranging from 30 to 500 nm (Figure 2a), indicating that nanoscale fiber networks were reserved in the decellularized matrix. The pig kidney scaffold still retained the fibrous structure after crosslinking. It was reported that the decellularized-derived matrix scaffold could provide more cell attachment sites, which was conducive to the adhesion and proliferation of cells on the scaffold, the conduct of various physiological activities, and the successful construction of the tumor model [37]. In addition, EDS analysis was conducted on different areas before scanning electron microscopy. The results showed that the pig kidney scaffold was composed of C (47.36%), N (15.41%), O (36.21%), and S (1.02%).

### 3.3. Physical Properties of the Pig Kidney Matrix Scaffolds

#### 3.3.1. Porosity of the Kidney-Derived Matrix

**Figure 3:**
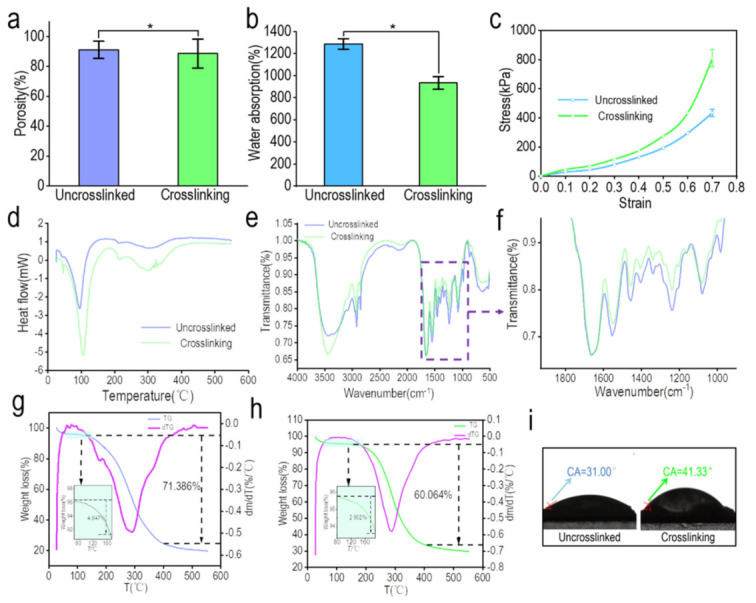
Physical performance analysis of pig nephritic-derived matrix scaffolds before and after crosslinking. (**a**) Porosity test of pig kidney matrix scaffolds before and after crosslinking, * *p* < 0.05. (**b**) Water absorption test of pig kidney matrix scaffolds before and after crosslinking, * *p* < 0.05. (**c**) Compression modulus test of pig kidney matrix scaffold before and after crosslinking. (**d**) Differential calorimetry scanning test of pig kidney matrix scaffolds before and after crosslinking. (**e**) Infrared spectrum detection of pig kidney matrix scaffolds before and after crosslinking. (**f**) Amplification of infrared spectrum at 1000~1800 cm^−1^. (**g**) TG analysis and TG differential curve of uncrosslinked pig renal matrix scaffolds. (**h**) TG analysis and TG differential curve of pig kidney matrix scaffolds after crosslinking. (**i**) Test of the contact angle of the pig kidney matrix scaffolds before and after crosslinking.

The porosity before and after the crosslinking of the pig renal matrix scaffold is shown in Figure 3a. The results showed that the porosity of the porcine kidney-derived matrix scaffolds decreased from 84.14 ± 3.20% to 81.09 ± 4.63% after crosslinking, which indicated a significant difference (*p* < 0.05) [38], As reported, dECM derived from animal tissues exhibited a porous structure with a porosity around 80%, allowing higher cell adhesion and cell invasion [13,39]. The decrease in porosity may be caused by increased molecular interaction after EDC/NHS intervention or the collapse and fusion of pores by secondary freeze-drying. This is also consistent with the observed slight decrease in the volume of the scaffold after crosslinking. 

#### 3.3.2. Water Absorption and Contact Angle of the Pig Kidney-Derived Matrix Scaffolds

Figure 3b,i shows the water absorption rate and contact angle performance of the pig nephritic-derived matrix scaffolds. Protein interactions and cell adhesion after scaffold transplantation depend on the scaffold’s ability to absorb water. In this study, the hydrophilicity of the pig kidney-derived matrix scaffolds was investigated by measuring the surface contact angle and water absorption of the scaffolds. There was no significant difference in the water absorption of the derived matrix scaffolds before and after crosslinking (*p* < 0.05), which decreased from 1287.39 ± 47.43% to 934.23 ± 55.65%. The results of the water contact angle showed that the contact angle of the scaffold increased from 31.00° to 41.33° after the surface of the scaffold was stabilized by deionized water. It may be that the crosslinking increases the hydrophobic property of the scaffold material and reduces the hydrophilicity of the scaffold, which is consistent with the result of the water absorption test. The decrease is due to the formation of amide bonds between the amino group and the carboxyl group, which reduces the number of hydrophilic groups on the molecular chain. After crosslinking, the molecular chains are closer together and the activities of hydrophilic groups are restricted, which prevents the scaffold from binding to water to a certain extent. However, compared with decellularized tissues from other animal sources, the crosslinked scaffold still exhibited a high hydrophilicity [39,40]. The microenvironment of native tissue is fluid, with nutrition, oxygen, and metabolic waste contacting directly with cells. Therefore, scaffolds with a higher hydrophilicity are able to absorb more nutrition in fluid medium and the exchange of metabolic waste is higher, promoting cell growth and proliferation [41]. 

#### 3.3.3. Compression Modulus of the Pig Kidney-Derived Matrix Scaffold

Figure 3c shows the compression modulus of the pig renal matrix scaffold before and after crosslinking. Biological scaffolds have certain mechanical properties and can bear certain loads in the process of tissue and cell growth since most cells tension is resisted by the matrix [38]. For many cell types, the tactile perception of matrix hardness feeds back into adhesion and the cytoskeleton, as well as net contractility [42]. Previous literature has shown that cell proliferation is rigid-dependent, with a greater ability to proliferate on hard surfaces [43]. The stress–strain curve of the scaffold was tested by a universal testing machine. According to the stress–strain curve, it is obvious that the crosslinking has a significant effect on the mechanical properties of the scaffold. At each strain level, the compression modulus of the scaffold increased significantly after crosslinking (*p* < 0.05). When the maximum strain was 0.7, the compression modulus of the crosslinked scaffold was 847.28 ± 58.05 kPa, and that of the uncrosslinked scaffold was 404.97 ± 22.63 kPa. As reported before, the compression modulus of native kidney is 41.62 ± 22.40 kPa, which is much lower than decellularized kidney [44]. Therefore, decellularization improved the mechanical characteristics of the kidney tissue, making it more flexible to load cell clusters.

#### 3.3.4. Thermal Stability of the Pig Kidney-Derived Matrix Scaffolds

The test results of the pig kidney-derived matrix scaffolds thermal stability are shown in Figure 3d,g,h. DSC-TGA was used to determine the thermal stability before and after the crosslinking of the pig kidney-derived matrix scaffold. It can be seen from Figure 3d that DSC presents a wide endothermic peak, indicating that the decomposition of the pig kidney scaffold is an endothermic process. With the increase of temperature, two obvious absorption peaks appeared near 95 °C and 300 °C, which correspond to the thermal shrinkage temperature and decomposition temperature of the material. The former is the temperature where the physical structure and folding structure of the material begin to be destroyed, and the latter is the temperature where the chemical bonds and hydrogen bonding within biological macromolecules begin to be destroyed. As the main component of the pig kidney matrix scaffold is collagen, a kind of protein, when the thermal shrinkage temperature is reached, the tertiary structure of the collagen molecule begins to dissociate and the microstructure is destroyed, indicating that the collagen become inactive. When the thermal shrinkage temperature is reached, the triple helix structure of the collagen molecule begins to dissociate and the microstructure is destroyed, indicating that the collagen is inactive. When the temperature rises to the decomposition temperature, the primary and secondary structures of collagen are completely destroyed and amino acids or other small organic molecules are formed. Therefore, the microstructure of collagen within the material was destroyed from 95 °C to 300 °C and it was broken down into small molecules. After crosslinking, the thermal shrinkage temperature of the scaffold increased to a certain extent as 12 ± 2 °C, indicating that crosslinking improved the thermal stability of the scaffold. 

In addition, the TGA curve is shown in Figure 3g,h. The figure also differentiates the TG curve to investigate the corresponding decomposition rate of the renal scaffold at different temperatures. The results show that during the whole heating process, there were two stages of weightlessness before and after the crosslinking of the scaffolds. In the first stage, the scaffold surface moisture and some small molecules were mainly lost, while in the second stage, the binding water in the scaffolds was mainly lost. At this time, the collagen molecules were completely destroyed and lost their activity. As binding water combined more tightly with chemical molecules within the material, it is more difficult to take binding water away, resulting in a slower weight loss in the second stage compared with the first stage. It is also consistent with the differential curve, which indicated the change of weight loss rate in Figure 3h. The decomposition rate of the scaffold first increased and then decreased, and reached the peak at about 290 °C, and the reaction ended at 400 °C. In terms of the weight loss rate of the pig kidney matrix scaffold, the weight loss rate of the uncrosslinked matrix scaffold between 80 °C and 150 °C was only 4.65%/°C and 71.39%/°C in the whole process; the weight loss rate of the crosslinked matrix scaffold between 80 °C and 180 °C was 2.90%, and the weight loss rate of the whole process was 60.06%/°C. It can be concluded from the weight loss rate that the crosslinked pig kidney scaffold maintains a good stability during the temperature rise compared with the uncrosslinked pig kidney scaffold.

#### 3.3.5. Infrared Spectra of Pig Kidney-Derived Matrix Scaffolds

Figure 3e,f shows the infrared spectrogram results of the pig kidney-derived matrix scaffold [45]. The main component of the scaffold is collagen, so the spectrum is mainly composed of the vibration of the amide group (amide band I, II, III, IV). Vibrational bands involved here are listed in Table 1, and the same absorption bands were observed in dECM as reported [46]. For the uncrosslinked scaffold spectrum, 3454 cm^−1^ was the N–H stretching vibration (hydrogen bond) peak of the amide A belt, while 2922 cm^−1^ was for the amide II band C–H stretching vibration peak. The spectra between 1000 cm^−1^ and 1800 cm^−1^ were further enlarged to obtain a clearer observation of the peak difference of pig kidney scaffolds before and after crosslinking [47]. The peak shape before and after the crosslinking was the same, but the transmittance was slightly different, indicating that the main chemical bonds within the scaffold were not changed after crosslinking, and the original molecular structure was still maintained. The difference of transmittance before and after crosslinking indicated that the C–H bond damage after crosslinking was greater than that before crosslinking, which may be due to the damage of secondary freezing to the scaffold. The strong peak at 1661 cm^−1^ was that of the amide I with the C=O stretching vibration, and also the COO-antisymmetric contraction vibration peak of the α helix. This peak may show a certain degree of red shift. The reason may be that the C=O group was affected by the hydrogen bonding C=O stretching vibration frequency or the coupling between C=O or C=O, which causes the frequency red shift. There was a peak at 1558 cm^−1^ for the amide II band with the N–H stretching vibration peak, while 1456 cm^−1^ was the bending vibration peak of -CH_2_- or -CH_3_-. Moreover, 1240 cm^−1^ was the stretching vibration peak of N–H, which belongs to the amide III band. The peak at 1080 cm^−1^ was the C–N stretching vibration or N–H stretching vibration peak of the amide IV band. For the scaffold after crosslinking, the amide I band from 1661 cm^−1^ to 1658 cm^−1^, amide II band from 1553 cm^−1^ to 1549 cm^−1^, amide III, and amide IV band did not change. In addition, the spectra between 1000 cm^−1^ and 1800 cm^−1^ were further enlarged to obtain a clearer observation of the peak difference of pig kidney scaffolds before and after crosslinking. In addition, EDC/NHS is a chemical crosslinking agent, connecting C=O and N–H to form an amide bond, which could also be a reason why the transmittance was lower after crosslinking.

Due to the limited mechanical properties of pig kidney matrix scaffolds, the chemical crosslinking method was adopted in this experiment to enhance the mechanical properties of the scaffolds [48]. Meanwhile, crosslinking also enhanced the thermal stability of the scaffolds, optimized the pore structure of the scaffolds, and made the original disordered pore structure become regular and orderly. In addition, IR spectra showed that crosslinking did not change the collagen structure of the scaffold. Moreover, the nanoscale fiber network is all over the scaffold, which is conducive to physiological activities such as cell adhesion and proliferation on the scaffold. In the following tumor model construction, a crosslinked renal scaffold is used for further experiments.

### 3.4. Biocompatibility of Pig Kidney Matrix Scaffolds

**Figure 4:**
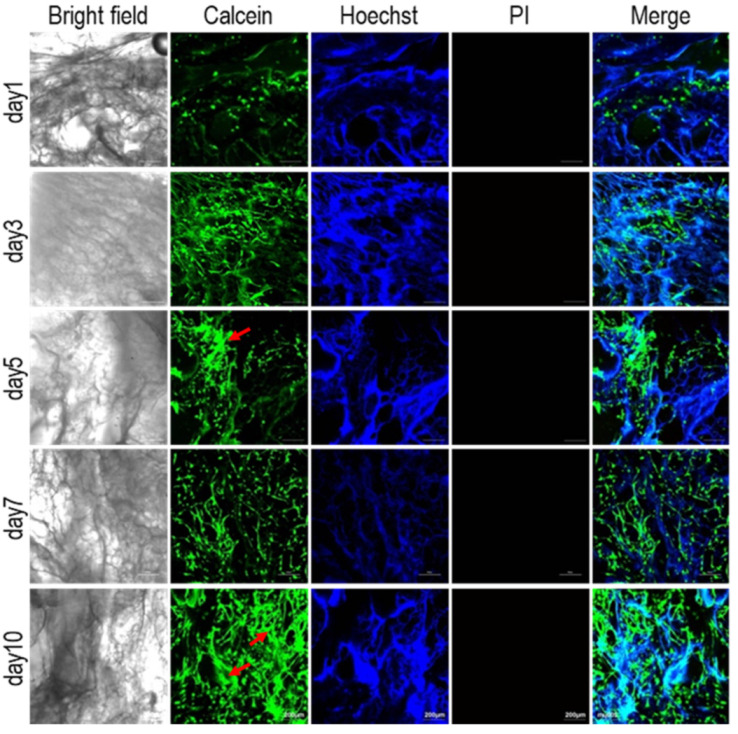
Growth of adipose tissue stem cells (ADSCs) on pig kidney-derived matrix scaffolds. The cells were dyed alive with calcein-Am, PI, and Hoechst 33,342, respectively; scale: 200 μm. The red arrows indicate cell colonies.

The biocompatibility test of the pig kidney matrix scaffolds is shown in Figure 4. The good biocompatibility of the scaffolds is essential for the successful construction of tumor models. Chaji et al. developed 3D tumor models based on 3D bioprinted hydrogels with ADSCs and MCF-7, as well as co-culture. It was found that MCF-7 exhibited a higher viability and metabolic activity than ADSCs [49]. Due to the infinite proliferation and high viability of MCF-7 cells, normal cells ADSCs were used to investigate the biocompatibility of scaffolds. Calcein-AM is a cell staining reagent that can fluorescently label living cells. The Hoechst marks all the nuclei and PI is often used for apoptosis detection. Collagen within the scaffold could also be stained by Hoechst, which allowed a comparison with the result of calcein-AM staining [50]. One day after inoculation, calcein-AM staining showed that the cell density was very low and a single cell adhered to the pore structure of the scaffold. Three and five days after inoculation, the number of cells gradually increased, and with the extension of time, the heads and tails of the cells slowly joined together. Seven days after inoculation, the cells gathered into colonies. Ten days after inoculation, the area of the colony became larger and larger as the number of cells increased (red arrow). Hoechst staining also revealed a gradual increase in the number of nuclei. No red staining cells (dead cells) were found by PI staining, indicating that the renal matrix scaffold has a good biocompatibility. 

### 3.5. Construction of the Breast Cancer Tumor Model

The establishment of a breast cancer tumor model will be evaluated from the following aspects: the adhesion of MCF-7 cells in 2D and 3D environments, the comparison of proliferation, the infiltration of MCF-7 cells in pig renal scaffolds, and the size of the tumor spheres.

#### 3.5.1. Growth of MCF-7 Cells in 2D and 3D Environments

**Figure 5:**
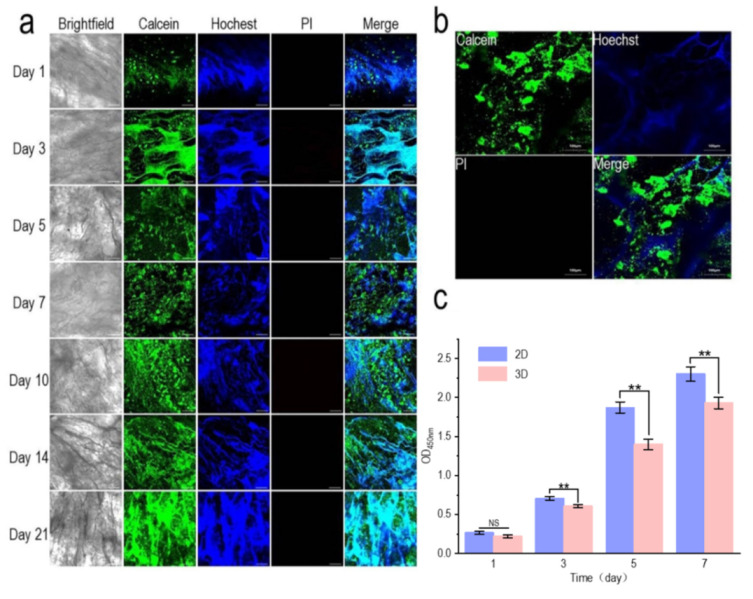
Growth of breast cancer (MCF-7) cells on pig renal matrix scaffolds. (**a**) MCF-7 cells were grown on the piglet renal matrix scaffold for 1, 3, 5, 7, 10, 14, and 21 days. The cells were dyed alive with calcein-Am, PI, and Hoechst 33,342, respectively; scale: 200 μm. (**b**) Dead and alive staining of MCF-7 cells on the pig renal matrix scaffold after 7 days growth, scale: 100 μm. (**c**) The survival of MCF-7 cells growing on 24-well plates and nephron-derived matrix scaffolds for 1 to 7 days was measured by CCK-8; NS, *p* > 0.05; ** *p* < 0.01.

The growth of MCF-7 on a pig kidney matrix was studied by live and dead staining (Figure 5). On the first day after inoculation, the cells were few and adhered to the surface of the scaffold as a single cell. On the seventh day, the scaffold was completely covered by cells. It is worth noting that the scaffold of an acellular pig kidney contains collagen, so the scaffold itself can be easily stained by Hoechst 33,342, but not easily stained by calcein-AM, especially when the number of cells is small and the scaffold is not covered in the first two days (Figure 5a). On the seventh day, certain parts of the scaffold with cells were enlarged, and it can be seen that the cells adhered to the void structure of the scaffold in a colony. From Hoechst 33,342 staining in Figure 5b, it can be seen that the scaffold was stained, but only the cells attached to the scaffold in the form of colonies can be seen in the calcein-AM staining. In addition, after 21 days of culture, calcein-AM and Hoechst 33,342 staining results also clearly showed the process of continuous cell proliferation. On the 21st day, the cells had already covered the whole scaffold (Figure 5a). CCK-8 was used to detect and compare the proliferation of cells on 3D acellular pig kidney scaffolds and 2D culture conditions (Figure 5c). On day 1, there was no significant difference in cell proliferation between the 3D scaffolds and 2D culture environments (*p* > 0.05). However, as the culture time was prolonged, the cell number difference was further increased from day 3 to day 7 (*p* < 0.01), with a significant difference. The reason why the cell proliferation in the 3D scaffold was lower than that in the 2D culture may be due to the different degrees of contact between the cells and the medium in the two culture systems. There is mass transfer inhibition in the 3D scaffold, which affects the transportation of nutrients, causing the cell proliferation rate in the 2D environment to be faster than in the 3D scaffold. In addition, the tumor spheres formed on the 3D scaffold had cavities similar to the necrotic tumor areas in vivo, which lacked oxygen, and the slow proliferation rate of tumor cells in the 3D culture system was also related to the hypoxic environment inside the tumor spheres [43], In addition, it was reported that cells grown on harder materials exhibit proliferation, while the microenvironment in vivo is softer than a solid engineered scaffold, so our tumor model stimulated a tumor microenvironment more similar to that of native tissues [51].

#### 3.5.2. Infiltration of MCF-7 Cells in Pig Renal Scaffolds

**Figure 6:**
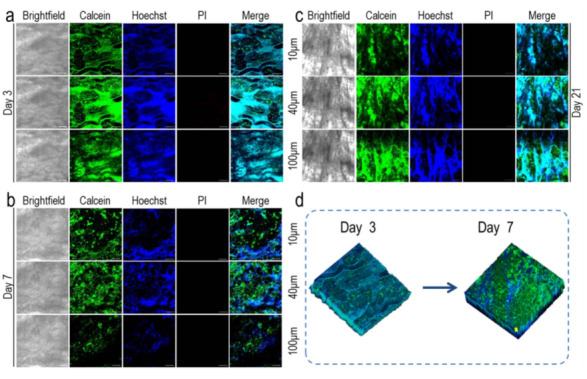
Infiltration of MCF-7 cells (10 μm, 40 μm, 100 μm) in pig renal matrix scaffolds by laser confocal microscopy. The cells were dyed alive with calcein-Am, PI, and Hoechst 33,342, respectively. (**a**) Infiltration of MCF-7 cells on pig renal matrix scaffolds after 3 days of growth; scale: 200 μm. (**b**) Infiltration of MCF-7 cells on pig renal matrix scaffolds after 7 days of growth; scale: 200 μm. (**c**) Infiltration of MCF-7 cells after 21 days of growth on the pig renal matrix scaffold; scale: 200 μm. (**d**) 3D reconstruction of 40 MCF-7 cells grown on a renal matrix scaffold for 3 and 7 days by laser confocal microscopy; scale: 200 μm.

The infiltration of MCF-7 cells on the renal scaffold is shown in Figure 6. The distribution of MCF-7 cells in scaffolds directly affects the development of tumor models in vitro. As reported, cancer cells exhibited eight hallmark capabilities including activating invasion/migration, which played an important role in tumor progression and the formation of a neoplastic state [52]. Del Bufalo et al. investigated cell invasion of a tumor model based on PEG-fibrin hydrogel by Matrigel invasion assay, confirming that tumor cells showed different levels of invasion under different culture conditions, and co-culture with HUVECs increased tumor migration. However, only the cell invasion at 24 h was investigated [53]. In this study, the distribution and infiltration of MCF-7 cells in pig renal matrix scaffolds during culture were investigated. After multi-layer scanning, the number of MCF-7 cells on different layers (10 μm, 40 μm, and 100 μm) was different, indicating that the permeability of MCF-7 cells in each layer of the pig kidney-derived matrix scaffold was different. Although it is not easy to stain the scaffold with calcein-AM, the morphology of the cells can be analyzed. It can be observed from Figure 6a that after 3 days of culture, most of the cells were located on the surface of the scaffold (10 μm). On the 7th day, the cells adhered not only to the surface (10 μm) but also inside the scaffold (40 μm), with a small number of cells osmotic at 100 μm (Figure 6b). The scaffold (10 μm, 40 μm, 100 μm) was covered with cells when the incubation period reached 21 days (Figure 6c). After focal length adjustment, the surface of the constructed material and its depth below 400 μm were sequentially scanned along the Z-axis, and the scanned images were further reconstructed in 3D. At day 3, calcein-AM infected few cells, but by 7 days of culture, the number of cells and the penetration depth of the cells were well reflected (Figure 6d).

#### 3.5.3. Investigation of Tumor Sphere Size

**Figure 7:**
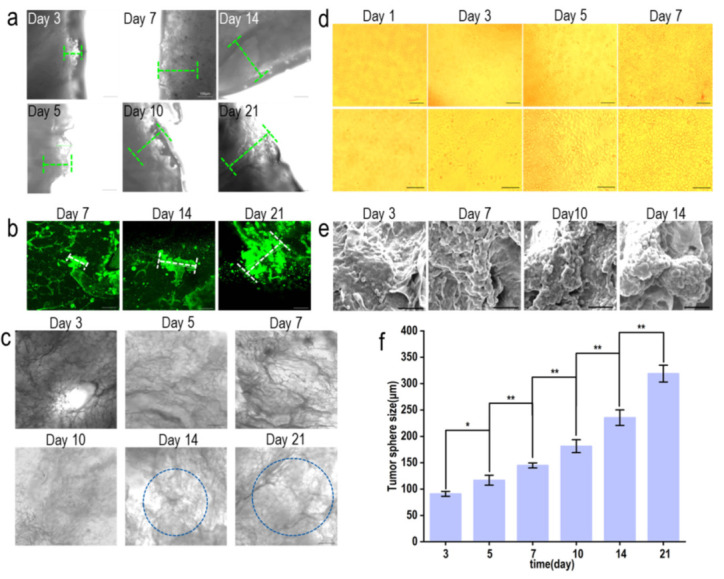
Tumor sphere size of MCF-7 cells after 3, 5, 7, 10, 14, and 21 days of renal matrix scaffold growth. (**a**) The tumor sphere size of MCF-7 cells at the edge of the scaffold was observed by inverted microscope 3, 5, 7, 10, 14, and 21 days after the growth of MCF-7 cells on the scaffold; scale: 100 μm. (**b**) Fluorescence microscopy was used to observe the size of tumor spheres inside the scaffolds after MCF-7 cells grew on the renal scaffolds for 7 days, 14 days, and 21 days; scale: 100 μm. (**c**) An inverted microscope was used to observe the overall distribution of MCF-7 cells cultured on the renal matrix scaffold 3, 5, 7, 10, 14, and 21 days later; scale: 200 μm. (**d**) MCF-7 cells were cultured on 24-well plates for 1, 3, 5, and 7 days with inverted microscope; scale: 200 μm (up), 60 μm (bottom). (**e**) Scanning electron microscopy was used to detect the size of tumor spheres of MCF-7 cells cultured on a renal matrix scaffold 3, 7, 10, and 14 days later; scale: 50 μm. (**f**) Combined with inverted microscope, scanning electron microscope, and laser confocal microscope images, IPP software was used to quantitatively measure the tumor sphere size inside and at the edge of the scaffold; * *p* < 0.05, ** *p* < 0.01.

The construction of the breast cancer tumor model is shown in Figure 7. Under an inverted microscope, the tumor sphere at the edge of the scaffold was observed to increase continuously, and the overall light transmittance of the scaffold was observed to decrease continuously (Figure 7a,c). Fluorescence microscopy was used to investigate the growth of tumor spheres inside the scaffolds, and the growth of tumor spheres inside the scaffolds increased from 7 to 21 days (Figure 7b). In the 2D environment, the cells proliferated rapidly, but no tumor sphere was found until the cells fell off from the scaffold (Figure 7d). After growing on the scaffold for 3 days, the first cells were observed to join into spheres (Figure 7e). The size of the tumor spheres at the edge of the scaffold and inside the scaffold was investigated. IPP software was used to measure tumor spheres after different culture days (n > 6): tumor spheres increased significantly from the third day to the fifth day (*p* < 0.05), and the fifth day, the seventh day, the tenth day, and the twenty-first day all showed a significant increase (*p* < 0.01). Scanning electron microscopy (SEM) and confocal laser microscopy (LSCM) images were used to quantitatively measure the size of tumor spheres inside and around the scaffold with IPP software. The results showed that tumor sphere size was increased from 90.85 ± 4.58 μm to 318.98 ± 15.98 μm from 3 to 21 days of culture (Figure 7f). This result was consistent with the report that different densities of cells resulted in different spheroid sizes, and the spheroid area of cells at the density of 45,000 cells/well turned out to be 6.0 × 10^6^ μm^2^ [54]. Reports showed that the size of tumor spheroids could be controlled at around 200 μm after 7 to 8 days when grown in microwells, which was the largest [55,56]. In this study, the size of the tumor sphere increased from day 3 to day 21, indicating that the platform simulated the process of tumor formation more realistically.

#### 3.5.4. HIF-1α and BRCA1 Expression in 2D and 3D Environments

**Figure 8:**
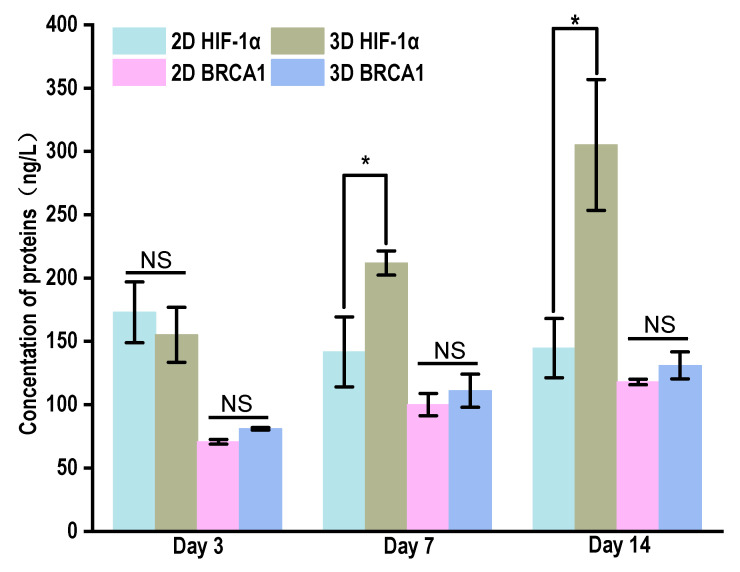
The expression of HIF-1α and BRCA1 in culture supernatant of MCF-7 cells in 2D and 3D culture system by ELISA. NS—not significant, *p* > 0.05; * *p* < 0.05.

The expressions of HIF-1α and BRCA1 in the supernatant of the cell culture after 14 days in the 2D and 3D environment were measured by ELISA (Figure 8). Hypoxia is common in many types of solid tumors, where tumor cells proliferate rapidly and form large solid tumor clumps, leading to blockages and compression of blood vessels around these clumps. Hypoxia plays a key role in the tumor microenvironment by upregulating vascular endothelial growth factor (VEGF) through hypoxia-inducing factor 1-α (HIF-1α). Upregulation of VEGE, in turn, promotes the production of new blood vessels, providing sufficient oxygen to tumor cells [57,58,59]. After renal scaffold growth, HIF-1α increased and HIF-1α expression was significantly different after 14 days compared with the 2D environment (*p* < 0.05), indicating that the renal scaffold can simulate the real tumor microenvironment in vivo. It was reported that the expression of HIF-1α varied with the thickness of the scaffold and inoculum density, and it could be detected using immunofluorescence at the depth of 150–200 μm [60]. In this study, it was consistent with the cell investigation of cell infiltration. BRCA1 is overexpressed in breast tissues, so it has typically been used as a marker to investigate the progression of breast cancer [61]. The expression of BRCA1 in the cell supernatant increased gradually under the two culture conditions. Although there was no significant difference, the densities of the 2D and 3D cells were the same. According to the above results, the proliferation of the 2D cells was significantly higher than that of the 3D culture conditions. Therefore, compared with the 2D environment, growth on the renal scaffold has more advantages in promoting the expression of BRCA1. Compared with the 2D growth environment, kidney-derived scaffolds can be more similar to the real tumor microenvironment.

## 4. Conclusions

We used porcine kidney-derived matrix scaffolds prepared by decellularization methods to construct close-to-real breast cancer tumor models. Porcine kidney decellularized matrix scaffolds can better simulate the tumor microenvironment in vivo with human cancer cells and promote cell growth and progression. Compared with the 2D culture system, although the proliferation rate of MCF-7 cells is slow, the cells in the pig kidney scaffold show an increased expression of breast cancer markers and hypoxic proteins. The tumor model constructed in this experiment provides a new method for studying tumor biology and treatment strategies. Future research will focus on considering cell types to improve the physiological complexity of breast cancer tumor models.

## Figures and Tables

**Table 1:** Main vibrational bands in infrared ray (IR) spectra before and after crosslinking.

Vibration Peak (cm^−1^)	Vibrational Bands
3454	N–H stretching vibration (hydrogen bond) of amide A
2922	C–H stretching vibration of amide II band
1661	C=O stretching vibration, COO- with α helix antisymmetric contraction vibration
1558	N–H stretching vibration
1456	CH_2_- or -CH_3_- bending vibration
1240	N–H stretching vibration of amide III band
1080	C–N stretching vibration or N–H stretching vibration peak of the amide IV band

## Data Availability

All data generated or analyzed during this study are included in this published article.
